# Inferences on mating and sexual systems of two Pacific *Cinetorhynchus* shrimps (Decapoda, Rhynchocinetidae) based on sexual dimorphism in body size and cheliped weaponry

**DOI:** 10.3897/zookeys.457.6512

**Published:** 2014-11-25

**Authors:** Raymond T. Bauer, Junji Okuno, Martin Thiel

**Affiliations:** 1 Department of Biology, University of Louisiana, Lafayette, Louisiana 70504-2451, USA; 2 Coastal Branch of Natural History Museum and Institute, Chiba, 123 Yoshio, Katsuura, Chiba 299-5242, Japan; 3 Facultad de Ciencias del Mar, Universidad Católica del Norte, Larrondo 1281, Coquimbo, Chile

**Keywords:** Fecundity, hermaphroditism, mate guarding, protandry, sexual selection

## Abstract

Sexual dimorphism in body size and weaponry was examined in two *Cinetorhynchus* shrimp species in order to formulate hypotheses on their sexual and mating systems. Collections of *Cinetorhynchus* sp. A and *Cinetorhynchus* sp. B were made in March, 2011 on Coconut Island, Hawaii, by hand dipnetting and minnow traps in coral rubble bottom in shallow water. Although there is overlap in male and female size, some males are much larger than females. The major (pereopod 1) chelipeds of males are significantly larger and longer than those of females. In these two *Cinetorhynchus* species, males and females have third maxillipeds of similar relative size, i.e., those of males are not hypertrophied and probably not used as spear-like weapons as in some other rhynchocinetid (*Rhynchocinetes*) species. Major chelae of males vary with size, changing from typical female-like chelae tipped with black corneous stout setae to subchelate or prehensile appendages in larger males. Puncture wounds or regenerating major chelipeds were observed in 26.1 % of males examined (*N* = 38 including both species). We interpret this evidence on sexual dimorphism as an indication of a temporary male mate guarding or “neighborhoods of dominance” mating system, in which larger dominant robustus males defend females and have greater mating success than smaller males. Fecundity of females increased with female size, as in most caridean species (500–800 in *Cinetorhynchus* sp. A; 300–3800 in *Cinetorhynchus* sp. B). Based on the sample examined, we conclude that these two species have a gonochoric sexual system (separate sexes) like most but not all other rhynchocinetid species in which the sexual system has been investigated.

## Introduction

The males of many animal species are larger in size than females and equipped with bigger or more specialized weapons (teeth, horns, claws, glands) used in fighting, competition for and defense of females. These observations were first meticulously documented by Charles Darwin (1871), who postulated that these male features were the result of an evolutionary process he termed “sexual selection”. Both female choice (epigamic selection) and intramale competition for females (or resources important to them) may be involved in sexual selection (Emlen and Oring 1977, West-Eberhard 1979, 1983, Thornhill and Alcock 1983, Tobias et al. 2012). Emlen (2008) has made an excellent survey and review of weaponry in a variety of animal taxa, summarizing the main hypotheses about selection pressures involved in their evolution.

Mating systems are behavioral strategies used by individuals of both sexes for finding and securing a mate for successful reproduction (Emlen and Oring 1977, Correa and Thiel 2003a, Bauer 2004). Sexual dimorphism in body size and weaponry in crustaceans, as in most other animal taxa, is highly correlated with mating system. Species in which males possess proportionately larger weaponry and body size than females are characterized by mating systems involving fighting for or defense of females, and/or maintenance of territories or other resources important for female reproductive success (Wickler and Seibt 1981, Grafen and Ridley 1983, Ridley 1983, Christy 1987, Correa and Thiel 2003a, Bauer 2004).

In decapod shrimps, sexual dimorphism in size and weaponry is closely associated with the mating system (Wickler and Seibt 1981, Correa and Thiel 2003a, Bauer 2004). In all penaeoidean and many caridean species, males are smaller than females and often live in mobile aggregations, e.g., the “schooling” species of important commercial fisheries. In these species (contrary to Ridley 1983), reproductive females do not advertise an upcoming molt, the time at which they are receptive to mating (all caridean, many penaeoidean species). When reproductive females of high-density species do molt, the mobile males soon find and mate with them via visual cues or contact chemoreception, as the frequency of contact with females is high. In this promiscuous mating system, termed “pure searching” by Wickler and Seibt (1981), males do not fight for or defend females (Correa and Thiel 2003a, Bauer 2004). Intramale competition takes the form of rapid and efficient searching for females. In other caridean species with “temporary mate guarding” or “neighborhood of dominance” mating systems, males are significantly larger than females and use hypertrophied weapons (major chelipeds, third maxillipeds) to fight for and defend females. In some species, such males are attractive to and sought out by females for mating (references in Correa and Thiel 2003a, Bauer 2004). Some caridean species live in pairs and are termed “monogamous” but their pair-living has been shown to be social monogamy (extended mate guarding) and not necessarily sexual fidelity (Knowlton 1980, Wickler and Seibt 1981, Mathews 2002). In such monogamous species, sexual dimorphism is slight compared to that of mating systems involving male guarding or defense of females (Bauer 2004).

The Rhynchocinetidae is a family of marine caridean shrimps found in subtropical and tropical habitats around the world. The common name “hingebeak shrimp” (McLaughlin et al. 2005) refers to the articulated rostrum, a unique character of the family (Chace 1997, Okuno 1997a,b). The family currently consists of two genera, *Cinetorhynchus* and *Rhynchocinetes*, with 11 and 14 species, respectively (Okuno 1997b, De Grave and Fransen 2011). The biological significance of the marked sexual dimorphism in male size and weaponry of *Rhynchocinetes typus* was first demonstrated by Correa et al. (2000, 2003). In a series of subsequent papers, the links among male size and weaponry, mating system and reproductive success have been extensively explored (Thiel and Correa 2004, Dennenmoser and Thiel 2008). In *Rhynchocinetes typus*, large “robustus” males are more successful than smaller more female-like “typus” males in copulating with and inseminating females. Females prefer to copulate with robustus males which, in addition to large size, are equipped with large chelipeds with a prominent setal tuft used for advertising sexual and size condition to conspecifics. Such males use these chelipeds to fight with other males over females. In *Rhynchocinetes typus*, the third maxillipeds of larger (“robustus”) males are hypertrophied, i.e., proportionately larger than those of females, and they are used as jousting weapons in male-male combat (Correa et al. 2003). These robustus characters develop ontogenetically from a female-like morphology in smaller males with an increase in male age and size (Correa et al. 2000). In another species, *Rhynchocinetes brucei*, Thiel et al. (2010) found a similar sexual dimorphism in weaponry although not so well developed as in *Rhynchocinetes typus*.

Examination of taxonomic species descriptions and personal observations (J. Okuno) have revealed that males with similar robustus males occur in some but not all rhynchocinetid species. Indeed, observations on *Rhynchocinetes uritai* from Japanese waters showed that this species is composed of small males and larger females without any sexual dimorphism in weaponry: male and female chelipeds and third maxillipeds are proportionately similar in size (Bauer and Thiel 2011). Unlike *Rhynchocinetes typus* and *Rhynchocinetes brucei*, the male mating system of *Rhynchocinetes uritai* is not one of “neighborhoods of dominance”, but rather appears to be a “pure searching” system. Furthermore, *Rhynchocinetes uritai*, unlike the other two species, is a protandric hermaphrodite, changing sex from male to female with increasing size (Bauer and Thiel 2011). Thus, there appears to be considerable variation in sexual dimorphism of body size and weaponry, and possibly sexual system, in the genus *Rhynchocinetes* and thus perhaps in its sister genus *Cinetorhynchus*.

Examination of taxonomic descriptions and personal observations by J. Okuno have revealed similar variation in the genus *Cinetorhynchus*. In this study, we explore this variation in two *Cinetorhynchus* species from Coconut Island, Hawaii, in which males exhibit a robustus-type morphology, i.e., large body size and hypertrophied major chelipeds. We analyze sexual dimorphism in body size and weaponry (major chelipeds, third maxillipeds) in these two *Cinetorhynchus* species and compare it with that previously described for *Rhynchocinetes* species. Morphometric data are used to test hypotheses on intramale competition and mating system in the two *Cinetorhynchus* species studied. We also test, with observations on relevant reproductive characters, the alternative hypotheses of gonochoric (separate sexes) versus protandric (sequential hermaphroditism) sexual systems in these species.

## Materials and methods

Two cinetorhynchid species were collected by M. Thiel on Coconut Island, Hawaii (19°43'46"N, 155°04'07"W) on March 16–20, 2011. Collections were made at night of these nocturnal species by dipnetting in shallow (wading) depths along the shore and spotting individuals by the reflection of their eyes under the light of headlamps. A few individuals were also captured with the aid of baited traps in somewhat deeper water (2–5 m). Specimens were preserved in 95% ethanol. Great care had to be taken with preservation and transport of males because their major chelipeds autotomize in life or detach in preservative very easily, and many males detached chelipeds in spite of careful handling.

There appeared to be two similar species, which were initially distinguished on the basis of overall body shape (“stout” and “slender”). Furthermore, from the morphological differences apparent in the structure of abdomen and antennular peduncle, the individuals were divided into two distinct species without doubt. But their available names under nomenclatorial rule have not been concluded. Therefore, in this paper, the “slender” one is referred to as *Cinetorhynchus* sp. A. [near *Cinetorhynchus hendersoni* (Kemp 1925)] and the stout species is termed *Cinetorhynchus* sp. B, closely related to *Rhynchocinetes intermedius* Edmondson, 1952, which is regarded as a junior synonym of *Cinetorhynchus hendersoni* (see Okuno 1997a). For reasons of manuscript readability, we refer to these two species as *Cinetorhynchus* sp. A and *Cinetorhynchus* sp. B in the following text.

Morphometric measurements on sexual dimorphism were made using a stereomicroscope with an ocular micrometer. A total of 14 males, 6 females of *Cinetorhynchus* sp. A, and 50 males, 14 females of *Cinetorhynchus* sp. B were examined, measured, and included in morphometric analyses. Specimens were sexed, using the presence (male) or absence (female) of an appendix masculina on the endopod of the second pleopod. The measure of body size, carapace length (CL), was taken as the chordal distance from the posterior edge of the eye orbit to the middorsal posterior edge of the carapace (e.g., Bauer 1986). As illustrated in similar rhynchocinetid species (Correa et al. 2000, Thiel et al. 2010: Fig. 1b), measurements on weaponry [major (first pereopod) chelipeds and third maxillipeds] were taken. The measure of major cheliped size was its propodal length, i.e., the distance from the proximal end of the propodus to the tip of the propodal finger (Thiel et al. 2010: Fig. 1b). For the third maxillipeds, the length of its distal segment (Thiel et al. 2010: Fig. 1b) and the number of dark corneous spines at its distal end (Thiel et al. 2010: Fig. 2c inset) were measured. Observations were made and recorded on the presence or absence of major cheliped injuries and regeneration.

In the course of measurements on the major chelipeds of males, an ontogenetic change in the chela to a subchela (McLaughlin 1980, Bauer 2004) was observed in which the propodal finger became increasingly reduced in larger males. To test the hypothesis of ontogeny in shape of the major chela in males, the length of the fixed (propodal) finger was measured as the distance from its base (a point corresponding to the basal articulation of the movable finger above) and its tip, and compared to total propodal length, the measure of major chela size.

Measures on two characters associated with incubation of embryos (‘‘breeding dress” in Höglund 1943, Bauer 2004) were taken on both sexes to investigate the possibility of male to female sex change. As figured for *Rhynchocinetes uritai* (Bauer and Thiel 2011), these characters were (a) the flange width of the basipod of the second pleopod (as in Bauer 1986) and (b) the height of abdominal pleuron 2, defined as the vertical chordal distance from the ventral edge of the pleuron to the middorsal line of the second abdominal somite.

Embryos were removed from incubating females with stage 1 or 2 embryos (as in Bauer 1991) (N = 2 in *Cinetorhynchus* sp. A and N = 7 in *Cinetorhynchus* sp. B) and counted. The lesser (*d1*) and greater (*d2*) diameters of five embryos, chosen haphazardly from each brood, were taken and measured, with medians used to calculate embryo volume [volume of oblate spheroid, *V* = 1/6 (π*d1^2^*d2)] (Turner and Lawrence 1979). Only embryos in early developmental stages (stage 1 or 2; see Bauer 1991) were utilized for measures of size and volume. The numbers of embryos (*Y*) were regressed against *X*, the body size (CL) of females. Log_10_ transformation was done on the dependent and independent variables whose values were plotted to calculate the slope in order to examine allometry of brood production with the equation “log *Y* = *b* (log_10_ *X*) + log *a*”, where *b* is the slope of the regression line and *a* is the y-intercept. The null hypothesis of isometry is *b* = 3, the slope of isometry for volume measures (brood volume as measured by number of embryos brood^-1^) plotted against a linear variable (CL) (Gould 1966).

## Results

### Sexual dimorphism in size and weaponry

**Figure 1.**
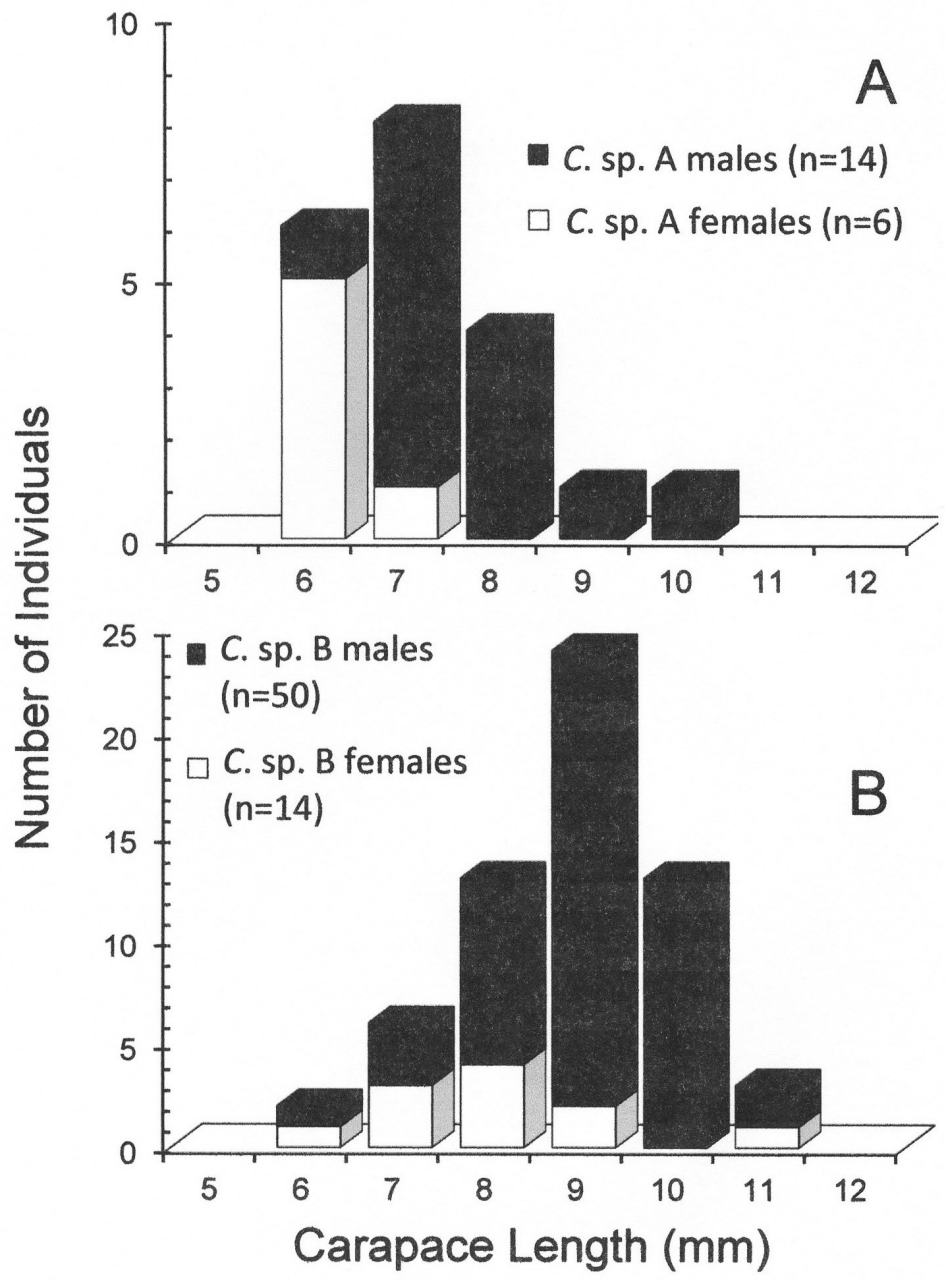
Size frequency diagrams of male and female *Cinetorhynchus* species. **A** *Cinetorhynchus* sp. A **B** *Cinetorhynchus* sp. B.

**Figure 2.**
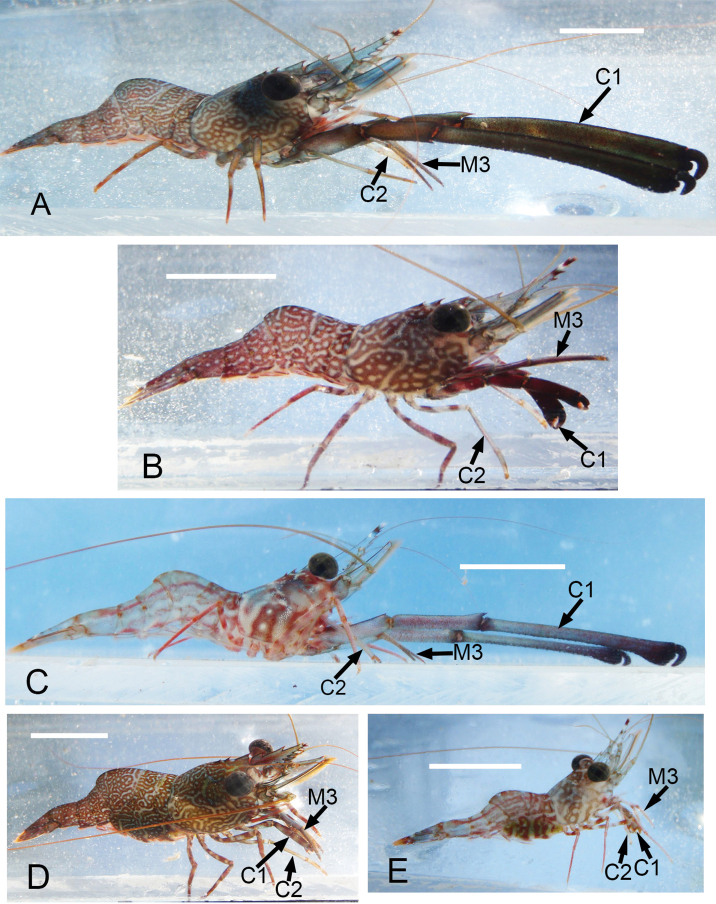
Photographs of living *Cinetorhynchus* species from Coconut Island, Hawaii. **A** *Cinetorhynchus* sp. B male with subchelate first chelipeds (pereopod 1) **B** *Cinetorhynchus* sp. B male with cheliped intermediate between chelate and subchelate **C** *Cinetorhynchus* sp. A male with subchelate chelipeds **D** *Cinetorhynchus* sp. B female **E** *Cinetorhynchus* sp. A female. **C1** cheliped 1; **C2** cheliped 2; **M3** third maxilliped. Scale bars represent 10 mm.

**Figure 3.**
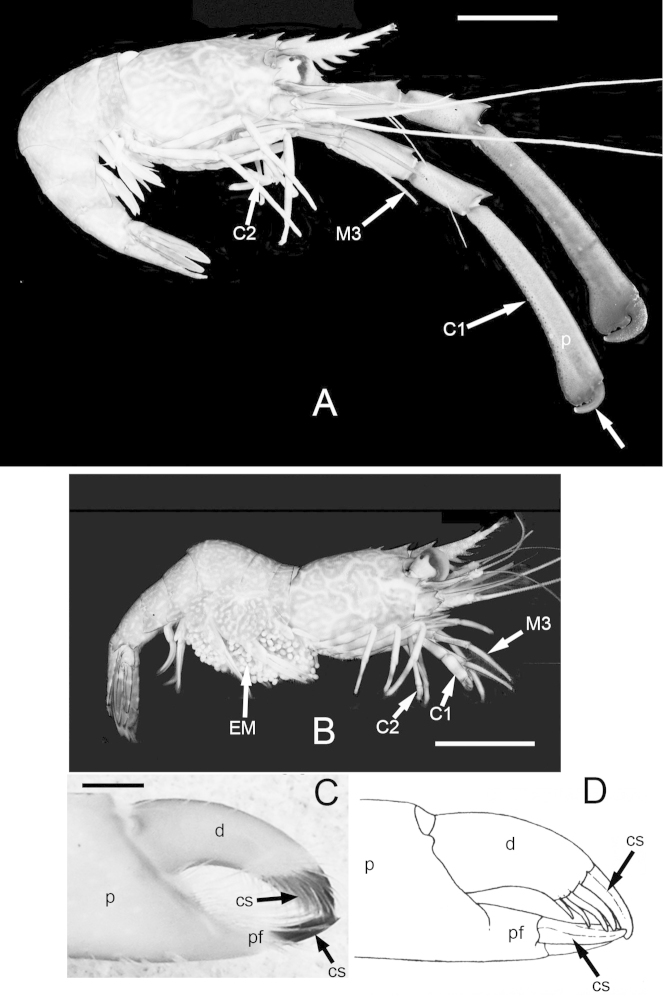
Sexual dimorphism in *Cinetorhynchus* sp. B. **A** Large male (10.8 mm CL) **B** Female (8.9 mm CL). Scale bars in A and B represent 10 mm **C** Distal end of chela 1 of a female (11.2 mm CL) showing blackened corneous setae on chela fingers; scale bar represents 0.5 mm **D** Illustration of tip of chela 1, *Rhynchocinetes albatrossae* (from Chace 1997, no scale given), showing form of blackened corneous setae (similar to those shown in Figure 3C) typical of chelipeds 1 and 2 of rhynchocinetids except in large males (e.g., as in this study). **C1** cheliped 1; **cs** corneous setae; **dactyl** (movable finger); **M3** third maxilliped; **p** propodus; **pf** propodal (fixed) finger.

**Figure 4.**
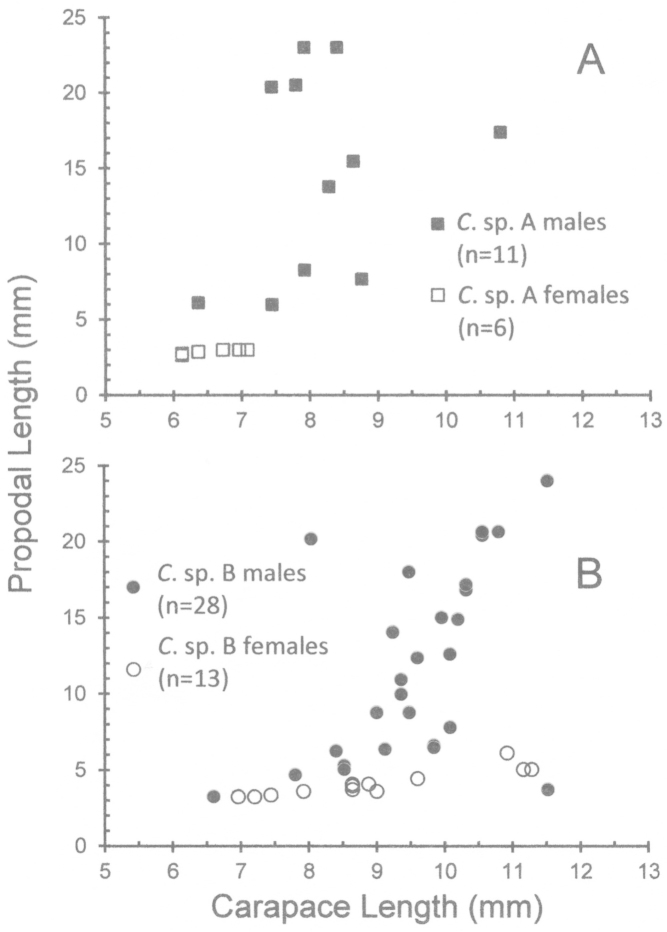
Variation of cheliped size in *Cinetorhynchus* species. Cheliped size (measured as propodal length) is plotted against body size (CL) in male and females. **A** *Cinetorhynchus* sp. A **B** *Cinetorhynchus* sp. B.

Although sample size is limited in our collection, it is apparent that in both species some males are markedly larger than most females (Figs 1–2, 3A–B). The size-frequency distributions of both species appear bimodal but only the intermediate and larger size classes were sampled by the collection (Fig. 1). Individuals of *Cinetorhynchus* sp. B reach a much larger size than those of *Cinetorhynchus* sp. A (Fig. 1A–B). Sexual dimorphism in cheliped weaponry is pronounced in both species. Males bear elongated, heavy major first chelipeds (pereopod 1) with elongated propodi in the larger males (Figs 2A–C, 3A, 4A–B). Additionally, chelipeds of larger males end in a wrench-like subchela (Figs 2A–C, 3A). Major chelipeds of females by comparison are small (Figs 2D–E, 3B, 4A–B) and terminate in a typical chela with equal fingers (Fig. 3B–D). In *Cinetorhynchus* sp. A, some of the larger males have proportionately much larger chelipeds than the smaller ones (Fig. 4A), while in *Cinetorhynchus* sp. B, the growth is more linear although with considerable scatter in the data (Fig. 4B). Female first chelipeds (pereopods 1) are shorter and not markedly robust (Figs 2D–E, 3B, 4A–B). As in all known Rhynchocinetidae (e.g., Holthuis 1993: p. 19, Okuno 1996), the chela fingers of both female pereopods 1-2, as well as male pereopods 2, terminate in several, stout, and black (highly sclerotized) setae (Figs 3C–D, 7A) which are reduced (Fig. 7B–C) or lacking in the major chelipeds of larger males (Fig. 7D–E).

**Figure 5.**
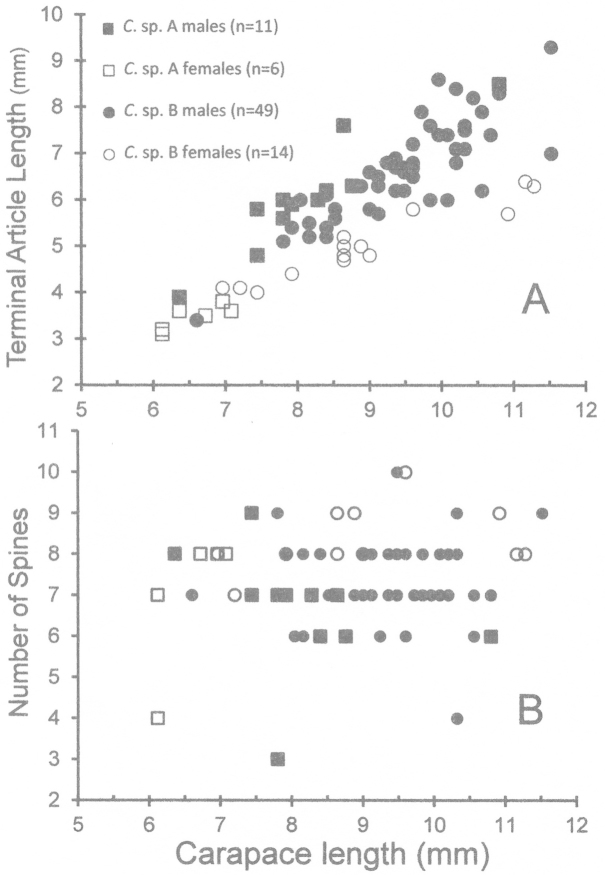
Comparison of third maxillipeds in male and female *Cinetorhynchus* species. **A** Maxilliped 3 size (measured as length of terminal article) plotted against body size (carapace length, CL) **B** Number of corneous spines on the terminal article of maxilliped 3 plotted against body size (CL). In **B** number of observations is the same as **A**, except for *Cinetorhynchus* sp. B males (n=45) and *Cinetorhynchus* sp. B females (n=13) because spinous portions of the terminal article of some individuals were damaged.

Two standard measures showing possible importance of the third maxillipeds as weapons (Correa et al. 2000, Thiel et al. 2010, Bauer and Thiel 2011) were compared between males and females of both species (Fig. 5A–B). The third maxillipeds of males in *Cinetorhynchus* sp. B (Figs 2A–B, 3A, 5A) are proportionately but not markedly larger than those of females (Figs 2D–E, 3B, 5A), which is also the case in *Cinetorhynchus* sp. A (Figs 2C, E, 5A), although the sample size of females in the latter is small (N = 6). In both species, there is considerable overlap between the sexes in the number of maxilliped 3 terminal spines (Fig. 5B).

### Ontogeny of major chelipeds in males

**Figure 6.**
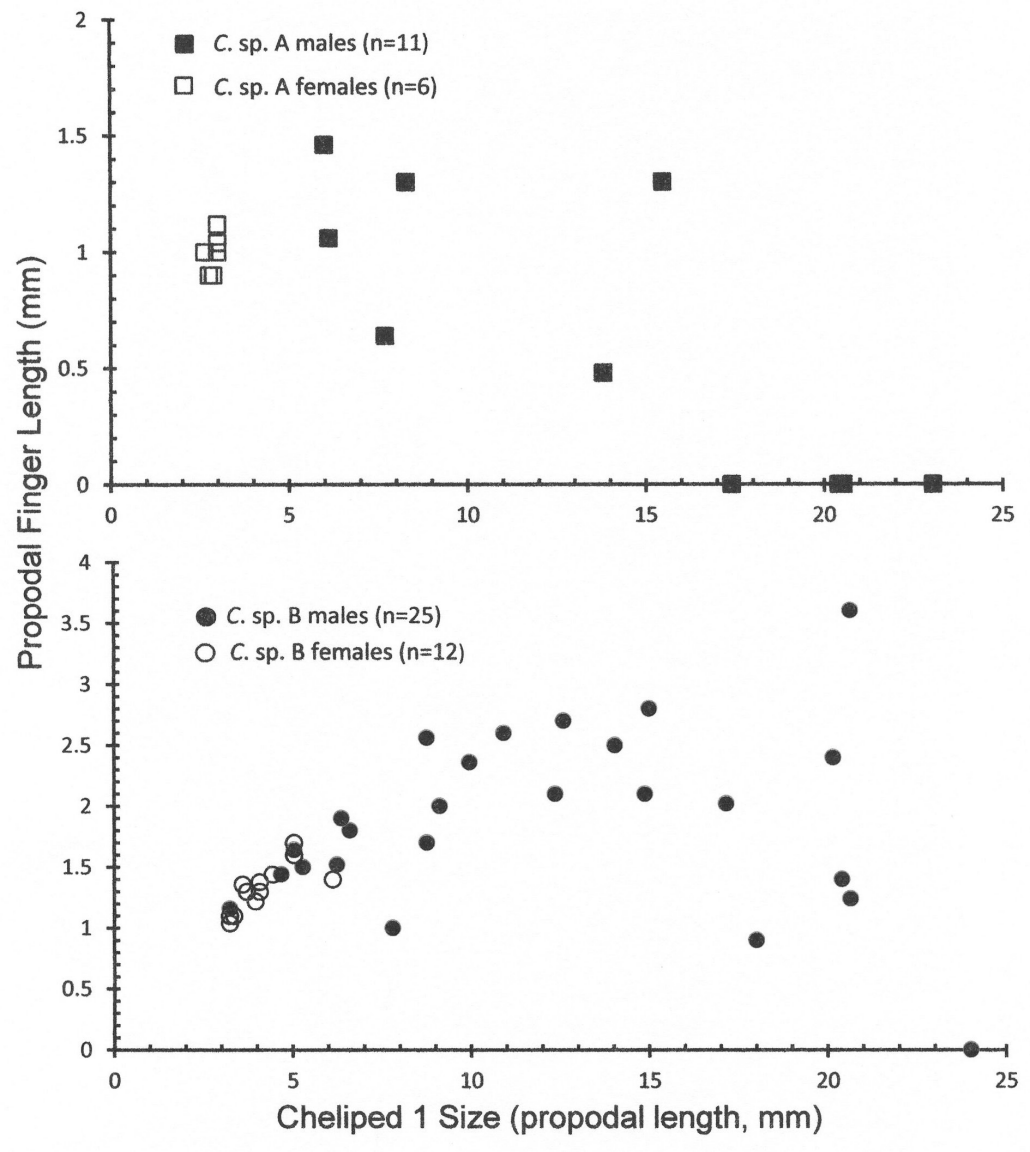
Ontogeny of major chela (pereopod 1) structure from a typical chela to a subchela in *Cinetorhynchus* species A and B. The length of the propodal finger relative to propodal length (cheliped size) is plotted as the measure of chela structure. With growth, the relative propodal (fixed) finger length decreases in larger males but not in females as male first chelipeds change from chelate to subchelate (see Figure **7**). **A** *Cinetorhynchus* sp. A **B** *Cinetorhynchus* sp. B.

**Figure 7.**
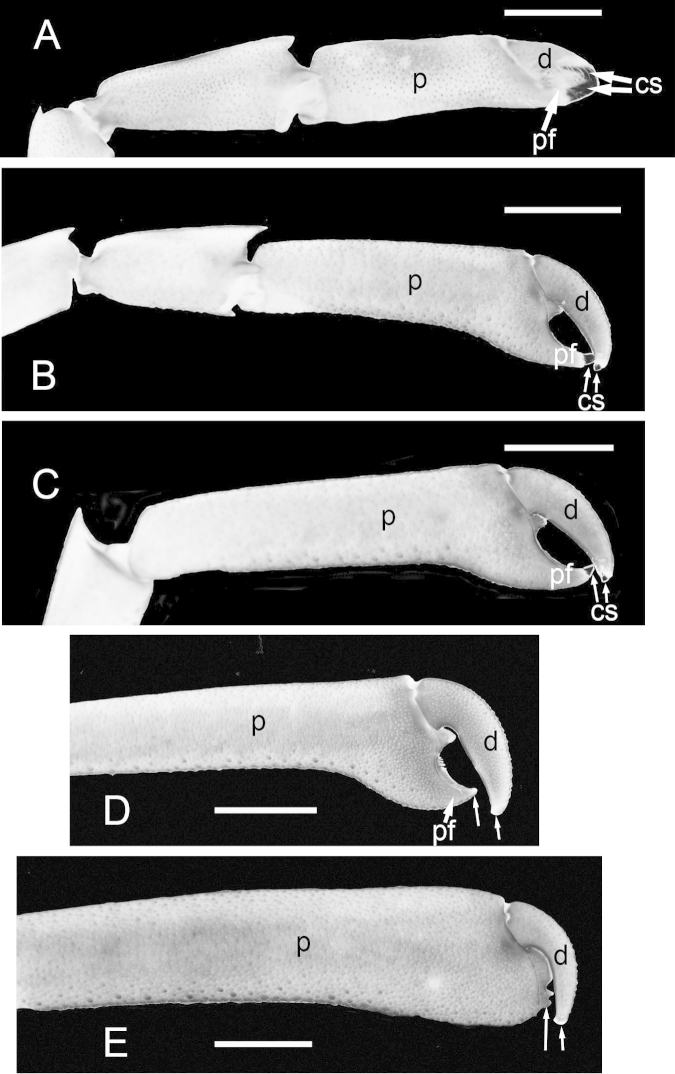
Variation in the major chela and chela finger shape with increasing size in males of *Cinetorhynchus* species B. **A** female 11.3 mm CL **B** male 9.4 mm CL **C** male 10.1 mm CL **D** male 10.6 mm CL **E** male 11.4 mm CL. **cs** corneous (black) seta(e); **d** dactyl (movable finger); **p** propodus; **pf** propodal finger. Unlabeled arrows in **D** and **E** show lack of the black corneous setae seen in **A–C**. Scale bars represent 10 mm.

With increasing size, males of both species show a major change in the shape of the chelae of the major cheliped (pereopod 1) while females do not (Fig. 6A–B). These changes were quantified by comparison of the chela’s propodal finger length vs cheliped 1 size, as measured herein by total propodal length (Fig. 6A–B). In males, there is a gradual change in the shape of the major chela from a true chela (with distinct upper movable dactylar and lower fixed propodal fingers that close together as in females, Figs 3B–D, 7A) to a more prehensile chela (Fig. 7B–D) to a subchela (Fig. 7E) in the largest robustus-type males. In females, chela 1 has equal propodal and dactylar fingers of a typical chela (Figs 3C–D, 7A), and is tipped with several black sclerotized (= corneus) setae (Figs 3C–D, 7A). In intermediate-sized males, the propodal finger is somewhat reduced compared to the dactylar finger (Figs 6A–B, 7B–D), and the setation becomes reduced in both fingers to a single black seta at the tip (Fig. 7B–C), which is lost with increasing size (Fig. 7D–E). In addition to decreasing propodal finger size with increasing chela 1 size, the distal end of the propodus also increases in height (Fig. 7B–E). The dactylar finger is flexed (Fig. 7E) against the expanded distal end of the propodus instead of closing against with a propodal finger as in females (Figs 3C–D, 7A), thus forming a subchela.

### Injuries to major chelipeds

**Figure 8.**
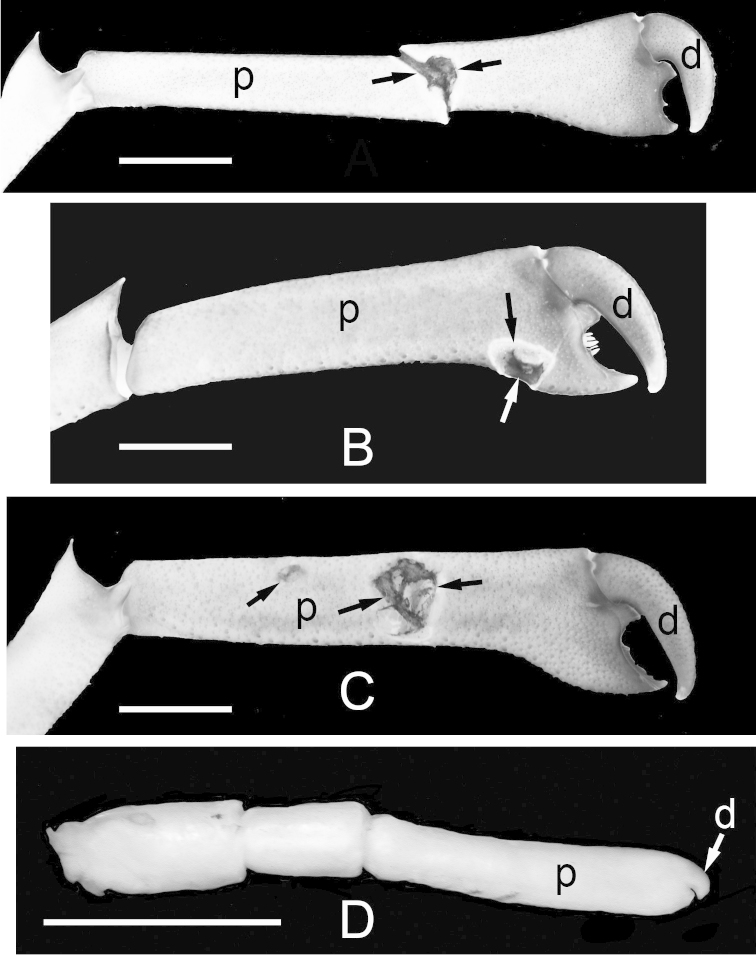
Puncture wounds (unmarked arrows) on the propodi of the major chelipeds of three large *Cinetorhynchus* males. **A** *Cinetorhynchus* sp. A **B** and **C** *Cinetorhynchus* sp. B **D** Regenerating major cheliped of a male *Cinetorhynchus* sp. B; only two articles plus a rudimentary cheliped with underdeveloped propodus and dactyl have formed. **d** dactyl (movable finger); **p** propodus. Scale bars represent 3 mm.

The major chelipeds of a number of males showed injuries. These injuries took the form of puncture wounds (Fig. 8A–C) to the elongated propodal portion (“palm”) of the major chelipeds. Previous possible injury, i.e., complete loss, of the cheliped may be indicated by regeneration of the cheliped (Fig. 8D). Puncture wounds were recognized by roughly circular perforations in the cuticle surrounded by melanized exoskeleton, a sign of scabbing and healing. Regenerating chelipeds were recognized by a combination of characteristics: much smaller than the other member of the pair (if present), a poorly sclerotized (whitish in preservative) cuticle, and a reduced number of poorly formed articles (Figure 8D). Of 38 males (both species) with one or both chelipeds, 10 individuals (26%) showed signs of injury (5 with puncture wounds, 5 with regenerating cheliped). By species, *Cinetorhynchus* sp. B males (N = 27), 3 (11.1%) had puncture wounds, while 4 (14.8%) had a regenerating major cheliped. In *Cinetorhynchus* sp. A males (N = 11), 2 (18.2%) showed puncture wounds and 1 (9.1%) had a regenerating cheliped. None of the females (*Cinetorhynchus* sp. A, *N* = 6; *Cinetorhynchus* sp. B, *N* = 14) showed any obvious sign of injury to their major chelipeds.

### Fecundity

**Figure 9.**
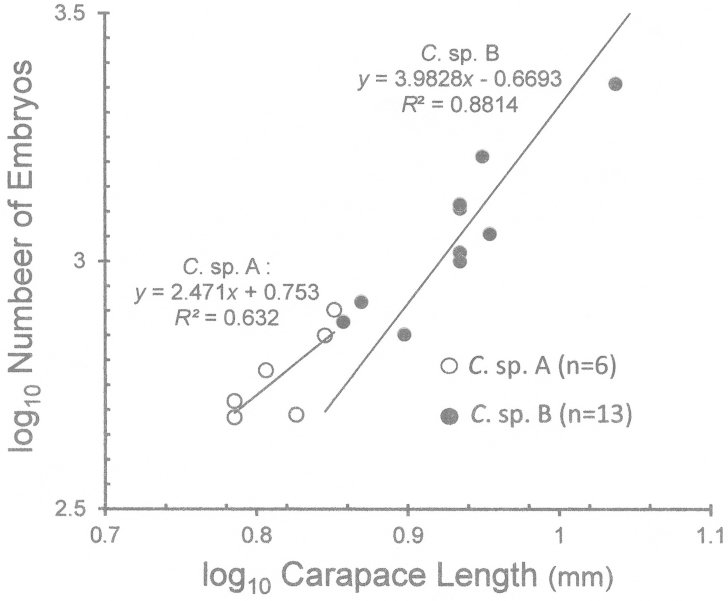
Fecundity in *Cinetorhynchus* species A and B. The log_10_ number of embryos per brood are plotted against log_10_ female size (carapace length, mm).

*Cinetorhynchus* sp. A had 484–798 embryos (*x* = 601 ±128, *N* = 6), while females of *Cinetorhynchus* sp. B carried from 304 to 3786 embryos per brood, (*x* = 1491 ± 104, *N* = 13). Linear regressions on log_10_-transformed variables (Fig. 9) indicated positive allometry in brood size (*b* > 3) in *Cinetorhynchus* sp. B (*b* = 3.983; 95% c.l.: 3.013–4.952). In *Cinetorhynchus* sp. A, *b* = 2.471, a number which could indicate negative allometry as *b* < 3. However, the 95% limits on *b* in *Cinetorhynchus* sp. A are so broad (-0.1469–5.090), probably due to small sample size (*N* = 6), that a definite statement on allometry in this species cannot be made. Females of *Cinetorhynchus* sp. A had slightly smaller embryos than females of *Cinetorhynchus* sp. B (Table 1) but the sample sizes are too small for a meaningful statistical comparison.

**Table 1.** Embryo size (*x* ± SD) of *Cinetorhynchus* species from Coconut Island, Hawaii, of females with stage 1 or 2 embryonic development. The medians of the lesser (*d1*) and greater (*d2*) diameters of 5 embryos brood^-1^, given below, were used to calculate embryo volume.

Species	*d1* (lesser diameter, mm)	*d2* (greater diameter, mm)	Volume (mm^3^)	N
*Cinetorhynchus* sp. A	0.37 ± 0.04	0.47 ± 0.07	0.034 ± 0.013	2 females
*Cinetorhynchus* sp. B	0.41 ± 0.02	0.50 ± 0.05	0.044 ± 0.007	7 females

### Breeding-dress characters and sexual system

**Figure 10.**
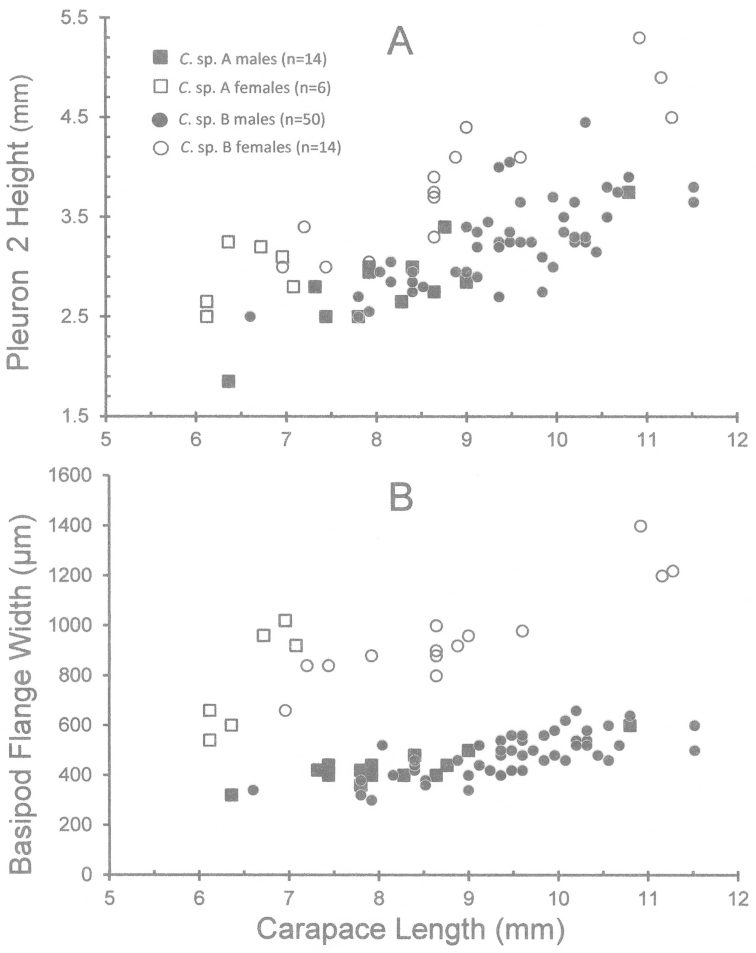
Variation in female breeding dress characters with increasing size in females in *Cinetorhynchus* sp. A and *Cinetorhynchus* sp. B. compared with the same structures in males. **A** Height of the second abdominal pleuron **B** Width of the basipod flange of the second pleopod.

To detect possible male to female sex change, two female breeding-dress characters were measured and compared between males and females: the height of the second pleuron (Fig. 10A) and the width of the basipod flange of the second pleopod (Fig. 10B). In both species the second pleuron is higher and the flange is wider in females than in males (Fig. 10A–B) throughout their size range, typical of gonochoric caridean species.

## Discussion

### Sexual dimorphism in size and weaponry

The sexual dimorphism in size and weaponry of the two Pacific species studied allows, for the first time, the formulation of hypotheses about the sexual and mating systems in the genus *Cinetorhynchus*. The population structure, composed of smaller females and larger males in the two *Cinetorhynchus* species, along with the presence of “robustus” males, i.e., large males with hypertrophied appendage weaponry, strongly suggests that the mating system involves some type of male-mate guarding. This type of mating system might vary from temporary (short-term) to extended (socially monogamous) mate guarding (Wickler and Seibt 1981, Bauer 2004) to perhaps the “neighborhoods of dominance” mating system found in species such as *Rhynchocinetes typus* and *Macrobrachium rosenbergii* (Correa and Thiel 2003a and references therein).

### Ontogeny of major chelipeds in males

The male weaponry of the *Cinetorhynchus* species studied consists of elongated, hypertrophied major (first pereopod) chelipeds bearing chelae whose form in larger males is more like a subchela than a chela. In a subchela, the movable (dactylar) finger folds against a vertically expanded distal end of the propodus (Bauer 2004). Males of at least 3 of 11 described *Cinetorhynchus* species, *Cinetorhynchus hawaiiensis* (see Okuno and Hoover 1998), *Cinetorhynchus hendersoni* and *Cinetorhynchus reticulatus* (see Okuno 1997a) also have hypertrophied major chelipeds. In females, the first chela is a “true” chela, i.e., the movable finger closes against a fixed (propodal) finger of similar length as in all rhynchocinetid species, which might be useful in autogrooming or grazing epibiota of hard surfaces. *Rhynchocinetes typus* is a known grazer and predator on fouling organisms of rocky and other surfaces (Dumont et al. 2011a,b). In males of the two *Cinetorhynchus* species, the second chelipeds are much like those of the females. In smaller males, the first chelae are somewhat female-like, but with increasing cheliped size, the sclerotized setae at the tip are lost, the distal end of the propodus expands vertically. The fixed (propodal) finger becomes proportionately shorter with an increase in cheliped size, so that in the larger “robustus” males, the first chelipeds bear a typical subchela. The loss of the possible grooming/grazing setae of the first chelipeds of robustus males may represent a cost associated with increased fighting ability. The considerable variation in cheliped-1 finger size in larger males might be due to loss of the cheliped in fighting and thus represents variation in the stage of regeneration and subsequent growth of the cheliped.

### Injuries to major chelipeds

The size and form of the male first chelipeds, as well as injuries to the first chelipeds (puncture wounds on the chela 1 propodus, regenerating chelipeds) in some males clearly suggests fighting between males. Presumably, such combat would be for access to or defense of females or resources attractive to them, as in many animals with hypertrophied weaponry (Emlen and Oring 1977, Emlen 2008). Rypien and Palmer (2007) found puncture wounds on the major chelipeds of a porcellanid crab, but these injuries occurred in both sexes and were more frequent in high density populations in wave-swept habitats than in low density and protected sites. The authors interpreted these data as evidence of both abiotic physical injuries and fighting among individuals of both sexes. Our observations on major male chelipeds injuries in the two *Cinetorhynchus* species are very similar to those of Rojas et al. (2012), who observed puncture wounds on the major chelae of males in the river shrimp *Cryphiops caementarius* (Palaemonidae) and reviewed examples from other decapods in which injuries are attributed to intramale fighting. Similar observations on damage in large males of *Rhynchocinetes brucei* were also interpreted as being caused by intramale fighting (Thiel et al. 2010). In the *Cinetorhynchus* species studied, we found no puncture wounds nor regenerating chelipeds in females, supporting the hypothesis that male injuries were a result of intramale combat. The hypothesis that the puncture wounds and regenerating first chelipeds observed result from fighting and not some other form of injury (predators, or physical injury by, for example, wave action in a coral-rubble habitat on elongated and exposed appendages) requires testing.

Hypertrophied weaponry is usually assumed and often has been shown to have evolved in many animals either because of intrasexual competition (fighting ability) among males for females and/or by female selection of males as potential mates based on their size and weaponry or other attractive characteristics (Darwin 1871, Emlen 2008). In *Rhynchocinetes typus*, robustus males do assess the fighting potential of other males when competing for females (Correa et al. 2003). In this species, males display their major chelipeds in the presence of receptive males by raising and lowering the major chelipeds. Furthermore, females are attracted to and chose among male suitors based on sexually dimorphic characters and behavior (Diaz and Thiel 2003, 2004). Sexual dimorphism in body size and weaponry in the two *Cinetorhynchus* species studied herein may similarly function in female selection of males.

The form of the subchela and the types of injuries found in the *Cinetorhynchus* species studied suggests the hypothesis that males may use their major chelae to grasp the long propodus of their opponent’s chelae, perhaps flipping or pushing them away from the female or refuge in which they are found. In *Rhynchocinetes typus*, if cheliped displays between similarly matched robustus males fail to resolve a contest over a receptive female, males fight by grasping each other’s major chelae and jabbing at each other with hypertrophied and spear-like third maxillipeds, often injuring each other (Correa et al. 2003). Although the third maxillipeds of the *Cinetorhynchus* species in this study were proportionately slightly larger than in the females, these appendages were similar in size and appeareance between the sexes, i.e., typical carideans third maxillipeds (see Bauer 2004). There was not the pronounced sexual dimorphism found in *Rhynchocinetes typus* and *Rhynchocinetes brucei* (Correa et al. 2000, Thiel et al. 2010). We propose that third maxillipeds of males in the two studied *Cinetorhynchus* species are not significant weapons as in *Rhynchocinetes typus* and *Rhynchocinetes brucei*. Such hypotheses in these two *Cinetorhynchus* species must await evaluation of larger samples over a longer temporal period, and especially for observations and experiments on living shrimps.

### Fecundity

Fecundity comparisons of these two *Cinetorhynchus* species with species of *Rhynchocinetes* (*Rhynchocinetes uritai* and *Rhynchocinetes typus*) suggest that fecundity is similar when body size differences are taken into account (Bauer and Thiel 2011, Vásquez and Castilla 1982, respectively). Our limited results suggest positive allometry in brood size (number of embryos with female size) in *Cinetorhynchus* sp. B and perhaps negative allometry in *Cinetorhynchus* sp. A. Early stage embryos were twice as large in size (an order of magnitude larger in embryo volume) than those reported for *Rhynchocinetes uritai*. Differences in embryo size and volume may indicate different fecundity/incubation period/larval development strategies among rhynchocinetid species that warrant further and more comprehensive study.

### Breeding-dress characters and sexual system

Males of these two species are as large or larger than females, a size frequency distribution which does not indicate protandric (male to female) sex change. However, given the finding of protandry in another rhynchocinetid species, *Rhynchocinetes uritai* (Bauer and Thiel 2011), other morphological characters indicating male to female sex change were analyzed. Key characters that indicate sex change are those involved in the reproductive female spawning/incubation chamber below the abdomen, i.e., the “breeding dress” (Höglund 1943). The height of the second pleuron and the width of the basipod flange of the second pleopod are two such characters (Bauer 1986, Bauer and Thiel 2011). In the *Cinetorhynchus* species examined here, there was no morphological shift in males to a female-like condition with an increase in size as one would expect in a sex-changing species. Thus, transitional individuals (sex changers) of intermediate external sexual morphology were not observed. The evidence presented here shows that these two *Cinetorhynchus* species, with large males bearing hypertrophied chelipeds, are gonochoric (separate sexes), as in two other such “large male” species investigated, *Rhynchocinetes typus* (Correa and Thiel 2003b) and *Rhynchocinetes brucei* (Thiel et al. 2010).

In both *Rhynchocinetes* (e.g., *Rhynchocinetes typus*, *Rhynchocinetes brucei*) and *Cinetorhynchus* (this study), the two current genera of the Rhynchocinetidae, there are species with large male size and hypertrophied male weaponry. A preliminary survey of the taxonomic literature on rhynchocinetids (J. Okuno, pers. obs.) indicates that there are also a number of species in the family in which males are on average smaller than females and without hypertrophied weaponry (authors pers. obs.). At least one species, *Rhynchocinetes uritai*, has a protandric sexual system (Bauer and Thiel 2011). Thus, this family may serve as an excellent model for testing hypotheses on the evolution of sexual and mating systems as well as sexual selection. The costs (e.g., higher exposure to injuries by predation, intramale combat, physical injury, energetic costs of growth) and benefits (access and/or attractiveness to potential mates) of hypertrophied sexual weaponry have been and continue to be a topic of considerable interest to evolutionary biologists (e.g., Darwin 1871, West-Eberhard 1979, 1983, Emlen 1983, Emlen 2008, Tobias et al. 2012). We suggest the family Rhynchocinetidae is an excellent system for such research. We will continue to expand our research on this family and invite others to join us in this venture.

## Conclusions

Sexual dimorphism in body size and weaponry strongly suggest a mating system involving male guarding or defense of females in the two studied *Cinetorhynchus* species. Some males are much larger than females in each species. The major weapons (pereopod 1 chelipeds) of males are significantly larger and longer than those of females. However, unlike some *Rhynchocinetes* species that have been studied, the third maxillipeds of males and females are similar in size in these two *Cinetorhynchus* species, i.e., do not appear part of male weaponry. Major chelae of males change with growth from typical female-like chelae tipped with black corneous stout setae in the smaller males to a subchelate or prehensile appendage in larger males. Puncture wounds on or regeneration of major chelipeds were observed in a number of large males. We interpret this evidence on sexual dimorphism and injuries as an indication of a temporary male mate-guarding or “neighborhoods of dominance” mating system, in which larger dominant “robustus” males fight for access and defense of reproductive females. The high overlap of male and female size and a lack of development of female breeding-dress characters clearly show that these two *Cinetorhynchus* species have separate sexes, unlike another rhynchocinetid (*Rhynchocinetes uritai*), which is a protandric hermaphrodite. Both the present and past studies on the Rhynchocinetidae indicate extensive variation in mating systems associated with differences in population structure and male weaponry, as well as variation in sexual systems. Thus, this family may serve as a good model with which to study the evolution of mating and sexual systems in other caridean families, as well as those of other animal taxa.
